# The Orthovanadate-Catalyzed Formation of a Thermally Inert and Low-Redox-Potential Melanin

**DOI:** 10.3390/ijms26125537

**Published:** 2025-06-10

**Authors:** Eric VanArsdale, Olufolasade Atoyebi, Okhil Nag, Matthew Laskoski, Evan Glaser, Eunkeu Oh, Gary J. Vora, Zheng Wang

**Affiliations:** 1 US Naval Research Laboratory, Center for Biomolecular Science & Engineering, Washington, DC 20375, USA; eric.s.vanarsdale.civ@us.navy.mil (E.V.); okhil.k.nag.civ@us.navy.mil (O.N.); gaurav.j.vora.civ@us.navy.mil (G.J.V.); 2 US Naval Research Laboratory, Chemistry Division, Washington, DC 20375, USA; sadeatoyebi@gmail.com (O.A.); matthew.laskoski.civ@us.navy.mil (M.L.); 3 US Naval Research Laboratory, Electronics Science and Technology Division, Washington, DC 20375, USA; evan.r.glaser.civ@us.navy.mil; 4 US Naval Research Laboratory, Optical Sciences Division, Washington, DC 20375, USA; eunkeu.oh2.civ@us.navy.mil

**Keywords:** biomaterial, biopolymer, eumelanin, selenite, vanadate

## Abstract

Catechol-like compounds are found throughout biology in the form of both redox-active and metal-binding functional groups. Within the marine environment, catechol groups are known to coordinate strongly with vanadate and ferric ions, and this binding is regulated through redox mechanisms. While investigating marine melanin formation in vitro, we found that DOPA, a catechol-containing amino acid, reacts with both metals differently when provided with sulfite, a weak reductant, and selenite, a weak oxidant. Both compounds interacted with the DOPA–vanadium complex, but only selenite, the more redox-labile chalcogenide, led to the creation of melanin particulates. When DOPA, vanadate, and selenite are present together, a metal-binding spectra shift and a melanin variant are rapidly observed. This variant was found to form large, elongated filaments with a low carboxylic acid content and a unique electron paramagnetic resonance signature. When compared to enzymatically produced melanin, this chemically synthesized variant was more thermally and biologically inert, exhibiting a lower redox activity. The results demonstrate that the regulation of the redox environment from metal–catechol interactions can help to control both the chemical and physical properties of melanin aggregates, suggesting a scalable and cell- and enzyme-free synthesis pathway for applications that may require inert materials of strict composition.

## 1. Introduction

The material properties of the ubiquitous melanin family of biopolymers derive from both physical and chemical realities [1,2]. Broadly, melanins comprise nano- to micron-sized aggregates that are formed from quinone-like substrates; they impart a functional redox moiety [3] and the capacity to strongly bind metals [4]. These properties, along with melanin’s notable broad-spectrum absorbance, are modulated by particle size, shape, and polydispersity, each of which are largely determined by the route of biosynthesis [5,6]. In nature, these properties are exploited by a myriad of organisms which structure melanins to create unique optical signatures [7] or selectively isolate their formation to prevent deleterious reactivities [8]. One salient example from the microbial world is that of fungal melanin, which has highly active quinone monomers but an overall low redox activity due to the decreased surface area-to-volume ratio of its cell-wall-embedded aggregates [9,10]. At present, the main synthetic route for tyrosine-based eumelanin is heterologous microbial production, which produces a black–brown pigment mixture of high polydispersity [11]. This process is cost effective and highly efficient [12,13], but creating tailored biomaterials for specific applications may require a more tightly controlled chemical environment [14]. With this in mind, we began to explore marine environments for unique melanin biosynthesis routes that could be regulated by novel strategies and scaled-for engineered material applications.

One well-studied marine metal–quinone interaction is that of orthovanadate (herein referred to as vanadate) with 3,4-dihydroxy-phenylalanine (DOPA)-modified peptides from the bivalve mollusk *Mytilus* spp. [15,16]. Vanadate is a weak oxidant that is captured by catechol groups within the thread-like anchors known as byssal threads, which the organisms use for surface attachment. This chemical complex is then transported to the cuticle in thiol-rich coacervates at low pH to disfavor metal-driven chelation and polymerization [17,18]. At the attachment site, the pH is increased, the thiol groups are oxidized, and the chelation and oxidation of the quinones results in strong attachment. A similar strategy is used by the vanabin-binding peptides in other marine invertebrates to sequester vanadate [19], which has been similarly demonstrated in artificial systems [20]. Using this natural mechanism as inspiration, we sought to apply the use of thiol-like interactions from sulfite and selenite to regulate vanadate-linked melanin formation in vitro [21].

## 2. Results

### 2.1. Selenite Modifies Vanadate–DOPA Interactions and Polymerization Rates

**Figure 1:**
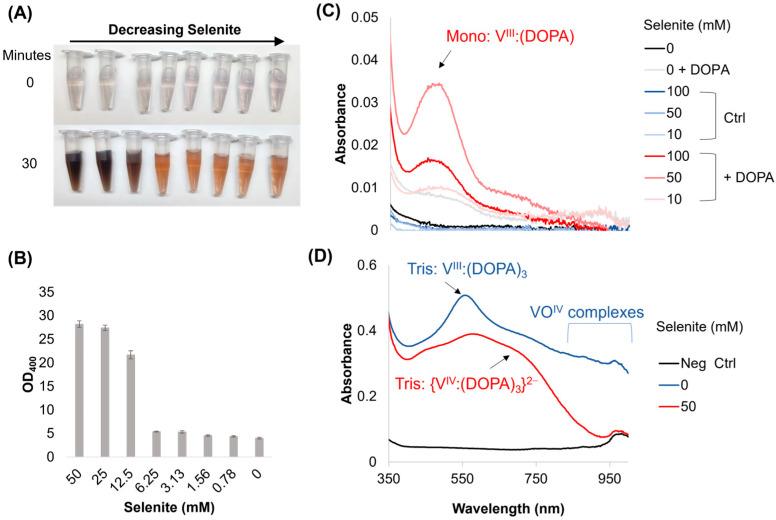
Melanin formation and UV-Vis spectra of vanadium–DOPA mixtures upon the addition of selenite. (**A**,**B**) Melanin is formed, without the enzyme, using 100 μM vanadate and 3 mM L-DOPA upon the addition of selenite. Values in (**B**) are measured after ~16 h. (**C**) UV-Vis spectrum scans of various reaction conditions upon the addition of 15 mM DOPA to 100 μM vanadate or (**D**) 1 mM vanadate with varying concentrations of selenite. The negative control (Neg Ctrl) = acetate buffer without any additives.

Selenite is a weak oxidant and a weak base, enabling two important aspects of melanin formation—(1) the oxidation and de-protonation of catechol into a quinone and (2) the subsequent oxidative polymerization of the resulting products. We first observed the ability of selenite to enhance vanadate-driven melanin formation in experiments involving vanadium-dependent halogen peroxidases (details in Supplementary Materials). In controls without enzyme, vanadate (the prominent vanadium species in seawater [22]) formed a light-purple color when added to a DOPA solution, although this quickly faded to a clear and then orange–red color, indicating dopachrome formation. However, when selenite was added to the mixture, the color shifted from purple to midnight blue (sub-panel Figure S1A), suggesting a vanadium redox transition from oxidation state +2 to oxidation state +4. The addition of selenite to the enzyme mixture at concentrations above 25 mM also noticeably increased the formation rate of dopachrome, measured at wavelength OD_492_, which is the colorful precursor of melanin (Figure S1A). Further characterization indicated that vanadate enhanced the chloroperoxidase oxidation of ABTS (Figure S1B) despite its reduction of hydrogen peroxide, a major key substrate, in the presence of all required cofactors (Figure S1C,D), but only at low enzyme concentrations. Therefore, we abandoned the enzyme and confirmed, via serial dilution, that selenite enhanced the oxidation of 15 mM DOPA through the addition of 0.1 mM vanadate in acetate buffer (0.1 M, pH 5.5, room temperature), which resulted in the formation of a colored product at concentrations above 12.5 mM selenite (Figure 1A), as well as the later formation of a melanin-like precipitate, measured via OD_400_, upon resuspension in deionized water (Figure 1B). Further investigation found that this reaction requires vanadate and did not proceed with sulfite (Figure S2A); both melanin formation and color change occurred only when the catechol/quinone species were used as substrates (Figure S2B). These results indicated that selenite interacted with DOPA–vanadate complexes in an interesting way, prompting further investigation.

We next used UV-Vis analyses to characterize shifts in the DOPA chelation of vanadate in response to selenite (Figure 1C,D). Peaks were analyzed using the literature as a basis (see Sever, M.J. et al. [23] for a more thorough discussion). When selenite was added to 0.1 mM solutions of vanadate with a saturating concentration of DOPA (~15 mM), we observed an increase in absorbance at ~470 nm, which we attributed to the mono chelate V^III^:DOPA, as well as a mild pH increase to 6.5 (Figure 1C). The absorbance at this peak increased over 15 min, along with a broad-shoulder increase at higher wavelengths, which could be a mixture of vanadium oxyanions (Figure S3A). We next increased the concentration of vanadate to 1 mM to increase the signal. The primary species of vanadium oxyanion in the starting solution should still be vanadate [24]; however, these tests do have a decreased DOPA-to-vanadate ratio. We observed that upon the addition of DOPA to the vanadate solution, there was an initial burst at ~560 nm, a smaller peak at ~738 nm, and a broad lifted shoulder at higher wavelengths, indicating a mixture of V^III^ and VO^IV^, the most predominant of which is tris chelate V^III^:(DOPA)_3_ (Figure 1D, blue curve). In comparison, when selenite was present at 50 mM, a Tris: V^IV^(DOPA)_3_^2−^ complex was observed (max peaks at ~580 nm and ~680 nm) and a small peak at ~470 nm appeared for the mono: V^III^:DOPA complex, while the higher wavelengths associated with VO^IV^ complexes were greatly attenuated (Figure 1D, red curve). Over time, the total spectrum of the selenite-treated sample continued to increase, with a further decrease in relative absorbance at ~470 nm and higher wavelengths in favor of the ~580 nm peak, while the sample without selenite appears to slightly flatten across the spectrum (Figure S3B). These spectra indicate that selenite increases the formation of higher-order chelates by preventing the re-oxidation of reduced V^III^ to VO^IV^.

**Figure 2:**
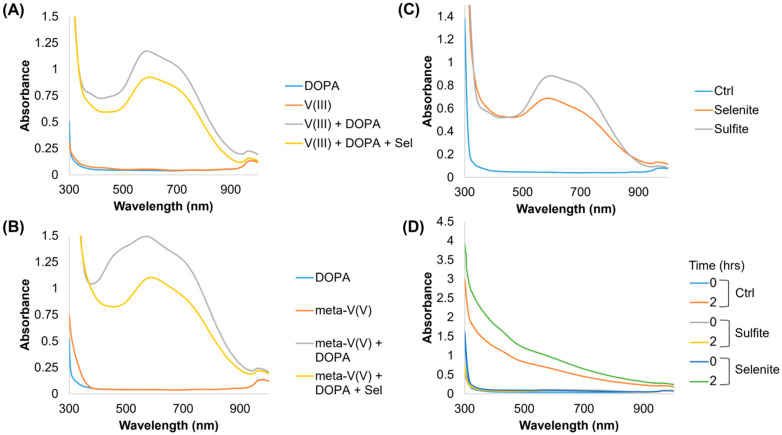
UV-Vis scans of alternative vanadium and thiol-like compounds. UV-Vis scans from 300 nm to 1000 nm for (**A**) 1 mM vanadium chloride (V(III)) and (**B**) 1 mM ammonium metavanadate (meta-V(V)) with 15 mM DOPA. (**C**) UV-Vis spectrum of 1 mM vanadate and 15 mM DOPA in the presence of 100 mM selenite or sulfite. (**D**) UV-Vis spectrum of 100 μM vanadate with 15 mM DOPA at 0 h and 2 h of incubation at 37 °C with 100 mM selenite or sulfite.

Previous work identified that the formation of a vanadate thioester during reduction with 3-mercaptopropinic acid was a rate-limiting step in the reduction of vanadate to V^III^ at a similar pH [25]. A similar reaction with selenite, which is more redox labile [26], could explain the drastic increase in V^III^ and V^IV^ as opposed to VO^IV^. In support of this, the same reactions were carried out using vanadium(III) chloride (i.e., not an oxyanion) and ammonium metavanadate (i.e., a different oxyanion). Selenite had no impact on the vanadium(III) chloride DOPA absorbance spectrum (Figure 2A), but it did shift the absorbance spectrum of ammonium metavanadate to more closely resemble higher-order coordination (i.e., the loss of a monovalent peak) (Figure 2B). Further, both selenite and sulfite initiate the formation of V^IV^(DOPA)_3_^2−^ when added to 1 mM vanadate solutions (Figure 2C); however, no melanin was formed over time within sulfite solutions (Figure 2D). We confirmed the same pattern of reactivity with Fe^III^ from ferric chloride; however, melanin formation was reduced (Figure S4A,B). Lastly, a cyclic voltammetry analysis of metal–DOPA–selenite right after mixing (Figure S5A,B) indicated that the addition of selenite modifies the reversibility of DOPA oxidation, and mildly shifts the DOPA oxidation potential due to an increase in pH.

### 2.2. Physical and Optical Characterization of the Novel Melanin Nanoaggregates

**Figure 3:**
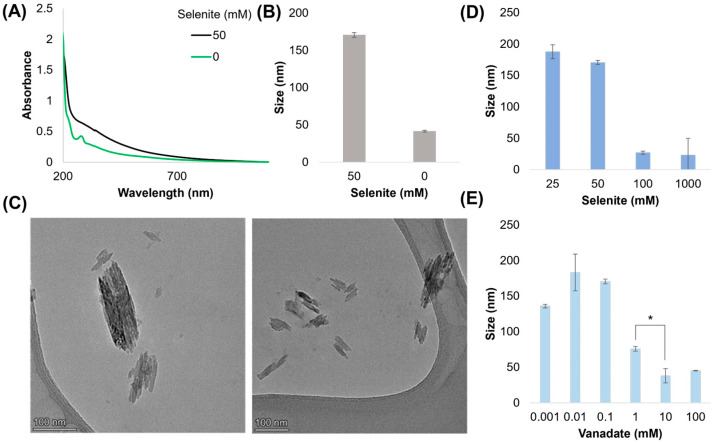
Morphological characterization. (**A**) Absorption spectra of purified and resuspended melanin produced with/without selenite. (**B**) DLS measurement of melanin samples with/without selenite. Melanin particles were not purified via acid–base cycling. (**C**) TEM images of nano-rods found in solution prior to purification. The long axis of the particles corresponded with DLS measurements. (**D**,**E**) DLS measurements (volume result) of nano-rod size when altering selenite or vanadate concentrations. * = significant difference (α ≤ 0.05).

Melanin is characterized by broad absorption across the UV-Vis spectrum. We compared the optical absorbance of 20× diluted melanin produced after 24 h with and without selenite (all samples comprise 1 mM L-DOPA, 100 μM vanadate) (Figure 3A), observing that the melanin formed with 50 mM selenite demonstrated modified binding in the UV range (<300 nm). Previous work has found that absorption in this region is sensitive to intermolecular interactions, such as decreased planar stacking [27], perhaps indicating that selenite is inducing chemical and structural changes that influence monomer interactions and eventual polymer formation. In comparing the dynamic light scattering (DLS) measurements of the volume-average size, we observed that the melanin samples formed with selenite contained larger radii of ~170 nm when compared to the ~41 nm particle size produced by the reaction of DOPA with vanadate alone (Figure 3B). Transmission electron microscopy revealed elongated, rectangular nano-rods with an uneven electron density, with the long axis corresponding to the DLS peak (Figure 3C). We were curious if these particles might be vanadium oxide [28] or vanadium–selenium compounds [29]; however, the particles disintegrated under higher beam voltages, making elemental analysis unreliable. When we increased the selenite concentration (Figure 3D), we observed that the particle sizes greatly decreased above 100 mM. Similarly, we saw the same effect when the vanadate concentration was increased (Figure 3E). A further analysis of particle size under biologically relevant conditions can be found in Figure S6A–D. Briefly, the results demonstrate that particle size (1) decreases with increases in pH, (2) increases with increasing salinity, (3) increases with temperature, and (4) greatly increases if pre-seeded. Of note, the V^IV^:(DOPA)_3_ complex has a charge of −2, which may contribute to charge repulsion among monomers, decreasing nano-rod size with increasing pH and low salinity. Both temperature and seeding suggest a potential aggregative growth or a form of nano-particulate ripening.

### 2.3. XPS Characterization of Purified Powder

We used XPS analyses to compare the elemental composition of auto-oxidized DOPA melanin as a control versus the vanadate-catalyzed DOPA–melanin produced with/without selenite. Each sample was characterized post acid–base purification (Table 1, Table 2 and Table 3). Representative XPS scans, peak fittings, and XPS peak binding energies each can be found in Figures S7–S9 and Table S1.

The elemental composition indicated that melanin produced with vanadate is closer to DHICA in terms of composition than the control, which is closer to DHI melanin [30] (Table 1). However, the addition of selenite drastically decreases the oxygen content of the resultant powder, which was unaffected by the addition of vanadate alone. There were also only trace amounts of selenium within the final powder, indicating that most of the selenium is not integrated into the DOPA backbone. Vanadate alone also reduced the nitrogen content relative to the base-oxidized DOPA powder. The ratio of these results indicates that vanadate-polymerized melanin has a higher oxygen/nitrogen ratio compared to the control, but the addition of selenite results in a lower oxygen/nitrogen content and a relatively greater carbon/oxygen ratio (Table 2). Elemental speciation shows that the major difference comes from the large increase in O-hydroxyl species in the selenite melanin at the expense of O-carboxyl groups (Table 3).

### 2.4. Redox and Electronic Characterization

**Figure 4:**
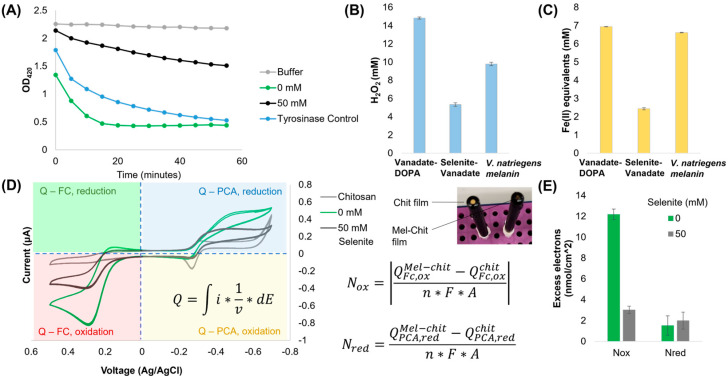
Redox characterization of melanin variants. (**A**) ABTS reduction by 0.1 mg/mL of each melanin variant. (**B**) H_2_O_2_ formation by 0.1 mg/mL of each melanin variant. (**C**) FRAP assay results using 0.1 mg/mL of each melanin variant. (**D**,**E**) Cyclic voltammetry analysis of 1 mg/mL of each melanin suspended in a chitosan film in 100 μM phenazine 1-carboxylic acid and 50 μM ferrocene dimethanol. Image in-lay shows a representative chitosan and melanin–chitosan film on standard gold-disk electrodes. Equations for the calculation of excess electrons transferred by each melanin sample are shown below the image in-lay.

Several chemical methods were used to characterize the redox properties of the novel melanins compared to a tyrosinase-catalyzed melanin produced by *V. natriegens* [12]. First, using the ABTS reduction assay [3], which is comparable to the food-standard Trolox assay, we found that the novel melanin made with selenite had an attenuated capacity to reduce ABTS, while the melanin produced with vanadate was more active than the tyrosinase control (Figure 4A). We next analyzed H_2_O_2_ formation from dissolved oxygen after an overnight incubation in PBS [31] and observed the same trends—the selenite melanin produced substantially less H_2_O_2_ than both the tyrosinase control and the melanin made with vanadate alone (Figure 4B). Lastly, we tested the reduction of Fe^III^ to Fe^II^ by each melanin using a ferric reducing antioxidant power (FRAP) assay. Again, we observed that melanin made with selenite has little ferric reducing power compared to the other melanins (Figure 4C). Previous experiments have attempted to standardize these measurements to Trolox equivalent antioxidant capacity (TEAC), or grams of Trolox per gram of melanin [32]. Using comparable relationships between the TEAC and FRAP evaluated previously [33], this would correspond to rough values of 1.12, 0.39, and 1.07 TEAC. Our DOPA control melanin is close to previous calculations of pure DHICA melanin (~0.9 TEAC [32]), thus illustrating the dramatic loss in redox activity for melanin produced with selenite and vanadate. However, we note that comparing between these experiments is challenging due to minor variations in experimental design.

There are two likely explanations for the reduced oxidative cpacity of melanin made with selenite and vanadate, as follows: (1) selenite removed key redox moieties from the melanin, and (2) physicochemical modifications to the melanin limit the available surface area. Of these considerations, it is important to note that vanadate-only (i.e., 0 mM selenite) particles are smaller and less polydisperse than previously produced tyrosinase melanin, as measured by DLS (single ~41.5 nm peak vs. >100 nm aggregates [13] with multiple peaks, respectively). Therefore, we believe that these results suggest that the vanadate-only sample has an enhanced redox activity due to its greater reactive surface area per gram relative to the control, while melanin made with selenite, conversely, has a significantly reduced redox activity due to its low surface area.

In order to further quantify the redox properties of these melanin variants, we created chitosan films with suspended melanin particles and subjected them to reverse electrochemical probing [10]. This method is semi-quantitative and calculates “excess electron” exchange relative to an inert control (i.e., an empty chitosan film). In this technique, two redox shuttling mediators are used (ferrocene dimethanol (Fc) and phenazine-1-carboxylic acid (PCA)) to pass electrons between melanin and the electrode during a voltage sweep. The charge accumulated from each mediator is then calculated using integrals in each quadrant centered at 0 V (see quadrants and cyclic voltammograms in Figure 4D), before being used in the equations listed for calculating excess electrons passed to the higher-potential mediator FC (*N_ox_*) and to the lower-potential mediator PCA (*N_red_*) (Figure 4E). We found that the melanin produced with vanadate alone passed nearly 6× more electrons to Fc per unit area of electrode compared to the melanin made with selenite. However, there was no significant difference in the electrons received from PCA for either variant. This indicated that both melanins are redox active, but selenite melanin has an attenuated oxidative capacity and therefore poorly interacts with reductants like PCA.

**Figure 5:**
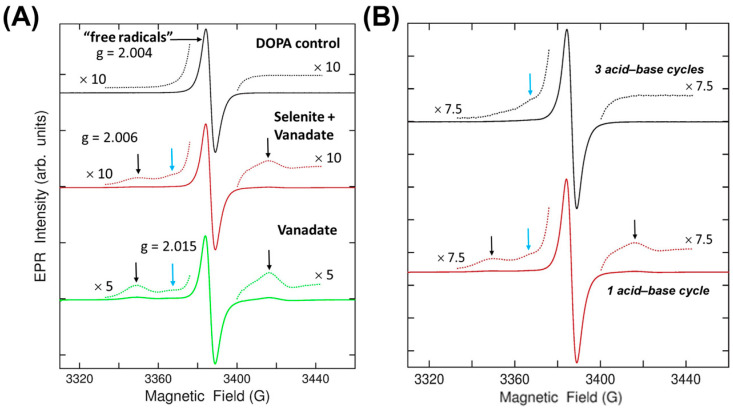
EPR characterization of reaction-purified melanin powders. (**A**) The EPR signatures of melanin powder made from DOPA (black; top), DOPA + selenite + vanadate (red; middle), and DOPA + vanadate (green; bottom). The magnification of the dotted lines is recorded next to each trace. The signature “free radical” signal’s corresponding g value is denoted above the top value, the small black arrows’ g value is labeled above the middle plot, and the small blue arrow’s g value is labeled above the bottom plot. (**B**) Comparison of EPR signatures from melanin made from DOPA + vanadate after three rounds of acid-base purification (top; black) or one round of purification (bottom; red).

We next measured the EPR signatures of purified melanin powder made from the base reaction products to further characterize the electronic nature of the melanin variants. The EPR signature of the DOPA melanin with a Zeeman splitting g-value of 2.004 is associated with a canonical “free radicals” peak that is commonly observed when analyzing eumelanin (Figure 5A). Melanin made with vanadate, as well as melanin made from vanadate and selenite, had this same peak. However, two new signatures of varying strength were observed in both powders. The two peaks labeled with the black vertical arrows each had a comparable intensity, suggesting it resulted from an S = 1/2, I = 1/2 vanadium-related complex with a g-value of 2.006, while the blue arrow indicated a peak with a g-value of 2.015 (of unknown origin). XPS measurements of the same powder did not detect any elemental vanadium. Therefore, the concentration of vanadium in these samples must be small but still higher than the EPR detection limit. Given that these powders were prepared using acid–base purification, we theorized that the vanadium-related signature might be labile to further purification. Indeed, after three acid–base purification cycles, the pair of lines labeled by the black arrows were diminished, while the weak signal labeled by the blue arrow remained (Figure 5B). These results indicate that vanadium was incorporated into the melanin during synthesis but can be removed by subsequent processing. Furthermore, there exists a unique EPR signature (blue arrow) that is created by vanadium and persists after its removal. No unique features were created by selenite.

### 2.5. Thermal Characterization Indicates Enhanced Stability

**Figure 6:**
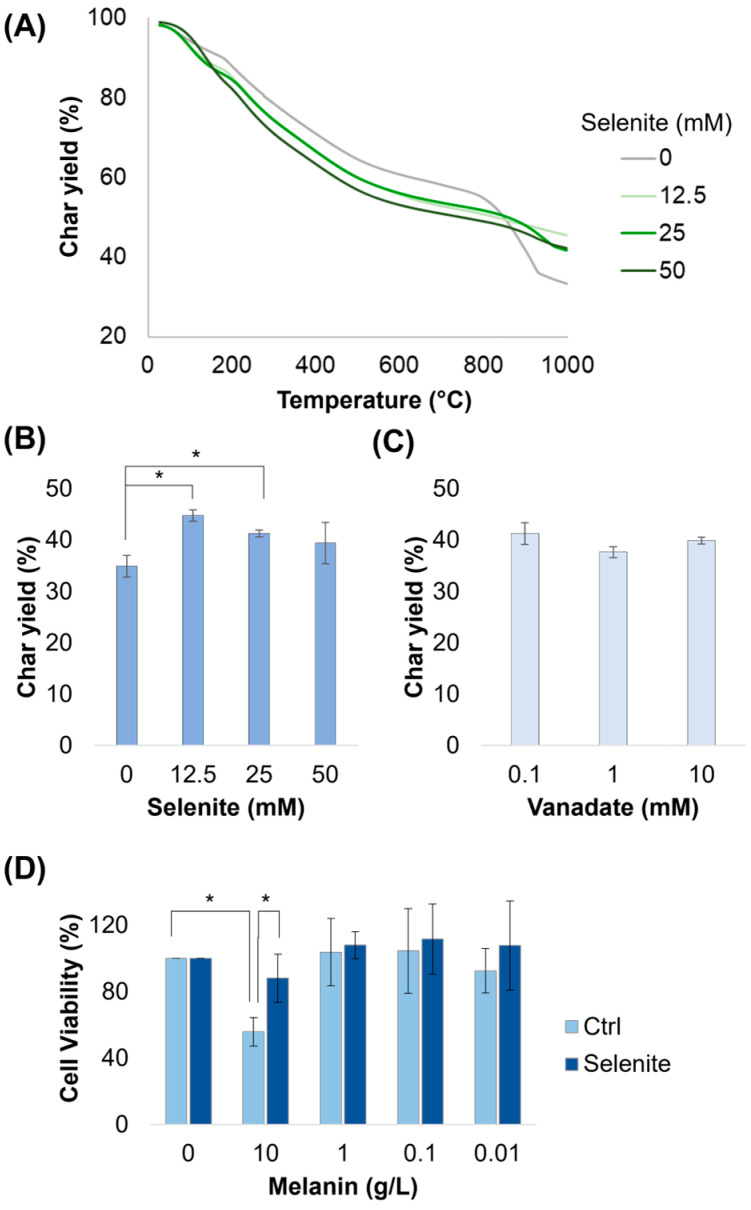
Thermogravimetric analysis and cellular toxicity of purified melanin variants. (**A**–**C**) Measurement of the thermal stability of purified melanin powders under a nitrogen atmosphere. Char yield is shown for melanin powder produced with various concentrations of selenite and vanadate. (**D**) HEK293 cell viability after a 2 h melanin treatment and 72 h recovery. * = significant difference (α ≤ 0.05).

Melanin has recently been investigated as a carbon additive for use in a high-temperature resin [34]. Here, we assessed the stability of the melanin variants using thermogravitometric analysis (TGA), during which the purified melanin powders were subjected to increasing heat under a nitrogen atmosphere. We found that all samples had similar response curves except for the final char yields, which were elevated in samples made with selenite (Figure 6A). The temperatures at which 5%, 25%, and 50% of the material weight was degraded, as well as the temperature of the maximum peak, are shown in Table 4. Oddly, this enhancement came almost exclusively due to the greater stability observed above 800 °C; on average, samples made with selenite lost more mass below 800 °C than that of melanin made without selenite, as was most evident in the T_5%_ and T_25%_ values. Increasing selenite concentration prevented accelerations in degradation at high temperatures; as such, samples made with 12.5 and 25 mM selenite saw peak degradation (T_peak_) occur at low temperatures, with a low rate overall. When comparing these samples, we observed that the highest char yield of ~44% was observed for the variant made with 12.5 mM selenite, which was ~10% higher than that without selenite (44.8% vs. 34.9%). Increasing the selenite concentration more dropped the char yield to 41% and 38% (Figure 6B). After repeating these experiments with 50 mM selenite in all samples, we found that vanadate did not improve char yield, even when increased by 100× (Figure 6C). These results demonstrate that this biomaterial has an enhanced thermal stability compared to melanin made without selenite. The enhancement is of about the same order of magnitude gained from the heat treatment of the melanin product [34]. Interestingly, we did not observe an increase in char yield when similar melanins were produced with selenite from catechol and dopamine—two melanin variants that do not have carboxylic acid moieties (Figure S10). Catechol and dopamine melanins did have a higher char yield than DOPA melanin, providing further evidence that the carboxylic acid moiety is a major driver of mass loss during TGA.

### 2.6. Reduced Toxicity of Melanin Variants to Cells

Catechol-functionalized polymers [31] and melanin [35,36,37] have been found to generate significant ROS upon illumination, in particular H_2_O_2_, in aerobic environments. In Figure 4B, we found significant differences in the amount of H_2_O_2_ produced by selenite–melanin in ambient conditions relative to a tyrosinase-produced control, suggesting it might therefore have a reduced impact on cellular life. We tested its toxicity towards mammalian cells using the CellTiter 96^®^ Aqueous One Solution MTS Cell Proliferation Assay and found that mammalian cells treated with 10 g/L of the tyrosinase control experienced significant toxicity compared to the null control; however, cells treated with melanin made with 50 mM selenite did not (Figure 6D). This indicated that this novel melanin, as measured by viability, has a reduced toxicity to mammalian cells.

## 3. Discussion

We have utilized a battery of physical, optical, redox, electronic, and thermal material characterization tools to reveal that DOPA oxidation by vanadate is modified by the addition of selenite, which drastically alters the final chemical composition of the resulting melanin. This occurs through a unique change to the DOPA–vanadate oxidation environment, which favors the formation of larger, lower-reduction-potential chelates, as well as a large reduction in the number of carboxylic acid moieties in the final aggregate. As a result, larger nano-rods were formed that had a reduced redox activity, an increased thermal stability, and a decreased toxicity.

Our current understanding of this reaction is as follows: vanadate is reduced through complexation with DOPA, de-protonating the catechol group under acidic conditions, leading to melanin formation, while selenite provides unique redox conditions to create a unique V^IV^:(DOPA)_3_ chelate by suppressing the re-oxidation of reduced vanadium into an oxyanion, thus encouraging the formation of Bis and Tris chelates. How selenite does this is unclear and beyond the scope of this work—what is clear is that it occurs via a thiol-like interaction, as evidenced by sulfite mimicking the initial effect. This unique V^IV^:(DOPA)_3_ species is proceeded by increases in the broad-spectrum absorbance in a time-dependent manner, likely due to the labile redox nature of selenium. This hypothesis is in agreement with previous studies of vanadium–catechol interactions [38], further demonstrating how thiol-like interactions can transition catechol oxidation even at low pH [17].

In terms of the physicochemical nature of melanin properties, the two largest deviations from melanin produced from the enzyme tyrosinase (i.e., eumelanin) included the dramatic increase in nanoparticle size and decrease in oxygen content from the loss of carboxylic acid moieties. In regard to the former, this large decrease in the surface area-to-volume ratio is at least partially responsible for the loss in redox activity, as less exposed surface is available to interact with the environment. This property is likely also related to the latter, as melanin made with selenite and vanadate had a low O-carboxyl speciation, which can prevent uniform monomer stacking. The loss of these moieties also appears to be specifically responsible for the improvements in char yield. This was also supported by the lack of improvement in the thermal stability of dopamine–melanin upon the addition of selenite, as well as the naturally greater TGA of dopamine–melanin relative to eumelanin, whereby dopamine lacks a carboxylic acid group. However, vanadate-catalyzed melanin oxidation alone more closely resembled 5,6-dihydroxyindole-2-carboxylic acid (DHICA) by elemental composition and was found to have potent redox properties compared to tyrosinase-produced melanin. Given that selenite removes the carboxylic acid from these particles, this may also be why, despite resembling DHI from a chemical perspective, the melanin produced by this interaction resembled the rod-like morphology of DHICA melanin when analyzed using TEM [39]. These results highlight the complex interplay between the physical and chemical variable space in melanin design and how subtle manipulations can result in large changes in material properties.

Importantly, these reactions demonstrated the efficacy of using thiol-like interactions to regulate biomaterial formation. We have previously demonstrated that this could be carried out to create reversible biomaterial formation [40,41], but this study demonstrates that they can also be used to control the oxidation rate of nearby chemicals. Here, selenite acts uniquely as both an antioxidant against dissolved oxygen, preventing vanadyl formation, and as a weak oxidant, forming dopachrome and melanin. This is consistent with the role of selenium in nature as a contextual, reversible redox agent [26]. In general, replacing sulfur with selenium in biomaterials is an interesting strategy to change oxidative properties [42] and reactivity [43]. Overall, the findings suggest that the careful planning of biomaterial synthesis around these thiol-like redox mechanisms can result in interesting new materials with a potent catalytic nature, or new material structures that present larger macromolecular properties.

In comparison to cellular or cell-free enzymatic synthesis methods, the chemical synthesis of DHI-like melanin is attractive for use cases that require inert materials of strict composition. For example, the novel melanin variant described in this study outperforms biologically produced melanin from *V. natriegens* [12,44] as an additive for improved thermal stability [34] due to the role of selenite during fabrication (which corresponded to the loss of the carboxylic acid moieties). The described melanin is also produced within a few hours, can be scaled to large volume production with minor modifications, and contains few impurities beyond salt, thereby eliminating the need for pre-treatment for thermal applications. We expect that most applications of melanin as material additives in filters [11], textiles [45], and coatings [46] could benefit from a distinct and controllable composition, which can be difficult, time-consuming, and expensive to achieve using enzymatic methods [39]. Similar benefits from inert materials could also have utility in medical applications, such as photothermal therapy [47] and magnetic resonance imaging contrast agents [48], where redox activity and impurities would compromise activity and safety.

## 4. Materials and Methods

### 4.1. Materials

All chemicals were procured from Sigma Aldrich (St. Louis, MO, USA) unless stated otherwise. *Escherichia coli* was grown in Lysogeny Broth (LB) (10 g/L tryptone, 5 g/L yeast extract, 10 g/L NaCl) for inoculation and maintenance, and toxicity was analyzed using M9 minimal salts medium (42 mM Na_2_HPO4, 24 mM KH_2_PO_4_, 9 mM NaCl, 19 mM NH_4_Cl, 1 mM MgSO_4_, 0.1 mM CaCl_2_, 20 mM glucose). *Vibrio natriegens* (strain ATCC 14048) was grown in LB supplemented with 300 mM NaCl for inoculation and transformation, and MM9v3 medium (80 mM K_2_HPO_4_ phosphate, 20 mM NaH_2_PO_4_ (pH 7.4), 50 mM NH_4_Cl, 5 mM Na_2_SO_4_, 275 mM NaCl, 1 mM MgSO_4_, 0.3 mM CaCl_2_, 20 mM glucose, 0.4% (*w*/*v*) glycerol, 0.2% (*w*/*v*) casamino acids, 0.2% (*w*/*v*) aspartate and 0.2× trace metals mix) was usd for the production of melanin, as previously described [12]. Electrodes were obtained from CH instruments (Bee Cave, TX, USA).

### 4.2. DOPA–Metal Interactions and Analysis

Reactions involving DOPA in the presence or absence of vanadate and selenite were performed in 50 mM acetate buffer,(pH 5.5) to prevent auto-oxidation. For the initial reactions that involved the vanadium-dependent halogen peroxidase from *Curvularia inaequalis* (ciVDHP), 1 mM potassium iodine and 1 mM hydrogen peroxide were present. In reactions involving multiple additions, vanadate was added first followed by selenite. The same order was used for reactions involving ferric chloride and sulfite (i.e., metal first, then supplement). For experiments involving UV-Vis, reactions were assembled in 96-well plates and scans were performed immediately upon the addition of the terminal chemical using a Tecan Spark Cyto plate reader (Tecan; Morrisville, NC, USA). In general, OD_492_ was used to measure dopachrome formation, while OD_400_ was used to measure general melanin formation. For transmission electron microscopy, a small reaction aliquot (5–10 μL) was drop-casted and dried on gold grids (01824G, TedPella, Inc.; Redding, CA, USA) and analyzed using a JEM-2200FS 200kV TEM (Jeol Ltd.; Peabody, MA, USA). DLS measurements were performed using a Malvern Zetasizer (Malvern Panalytical; Westborough, MA, USA) at room temperature with a 90° angle and sizes, which were averaged over three measurements, were reported according to the volume result.

### 4.3. Melanin Purification by Acid–Base Cycling

The melanin product was purified through acid precipitation and washing with water, as previously described [12]. Briefly, melanin was precipitated from reactions for 30 min by the addition of 6 N HCl and then pelleted by centrifugation at 15,000 rcf for 15 min. When multiple cycles were performed, 1 N NaOH was used to resolubilize the melanin prior to acid re-precipitation. After discarding the supernatant, the pellet was re-solubilized with water to remove excess salt. The melanin was then re-pelleted by centrifugation and washed 3× with water. Lastly, the pellet was then left to dry in a 60 °C oven for at least 24 h. The result was a dark-black pellet of granules. This pellet was then ground with a mortar and pestle to create a fine black powder for further testing.

### 4.4. XPS Analysis and Curve Fitting

X-ray photoelectron spectroscopy (XPS) analysis was performed as previously described [34]. Briefly, analyses were performed using a Nexsa G2 XPS (Thermo Scientific; Waltham, MA, USA) fitted with an Al Kα monochromator, generating X-rays calibrated at 1486.6 ± 0.2 eV. Survey and high-resolution analyses were performed at pass energies of 200 and 20 eV, respectively. All data were collected using a flood gun emitting Ar^+^ ions, for charge neutralization, raising the pressure in the analysis chamber to no greater than 5 × 10^−8^ mBar. All samples were analyzed in triplicate, each spot roughly 400 µm in size, and analyzed using Avantage software (version 5.9).

### 4.5. Characterization Assays

#### 4.5.1. ABTS Reduction Assay

The ABTS reduction assay [3], which is comparable to the food-standard Trolox assay, was performed by electrochemically oxidizing ABTS (0.7 V, 1 h) to a bright-green color (max. absorbance ~420 nm), before adding 0.1 mg/mL of melanin powder. Each sample was then optically monitored at 420 nm every 5 min for 1 h.

#### 4.5.2. Hydrogen Peroxide Formation Assay

For each sample, 0.1 mg/mL of melanin powder was dissolved in 1× PBS and incubated with shaking at 37 °C overnight. H_2_O_2_ was then measured from each sample using a Pierce assay (Thermo Fisher; Waltham, MA, USA) and compared to a standard curve produced with dilutions of 30% H_2_O_2_.

#### 4.5.3. Ferric Reducing Antioxidant Power (FRAP) Assay

The FRAP assay from Abcam (Waltham, MA, USA) was used to analyze purified melanin powder. Briefly, 0.1 mg/mL was added to 1 mL of each reaction and incubated for 1 h with shaking. Absorbance was then measured and quantified versus a standard curve, as per the manufacturer’s instructions.

### 4.6. Electrochemical-Mediated Probing

For electrochemical testing, melanin–chitosan films were prepared on gold electrodes as previously described [10] with slight modifications. Briefly, a 0.5% chitosan solution (pH 5.5) was mixed with melanin powder to a concentration of 1 g/L. A total of 20 μL of the solution was then cast onto the electrode and was then vacuum dried for 15 min. The film was then neutralized in phosphate buffer (0.1 M; pH 7.5) for 10 min. Empty chitosan films were used as a control. Films were probed using 100 μM phenazine-1-carboxylic acid (PCA) and 50 μM ferrocene dimethanol (FC) as our reductive and oxidative mediators, respectively. Cyclic voltammetry sweeps were performed with modified gold electrodes in the previously described set-up between −0.65 and +0.5 V vs. Ag/AgCl at a scan rate of 5 mV/s. All measurements were performed using a CH Instruments 720E potentiostat (Bee Cave, TX, USA).

### 4.7. Electron Paramagnetic Resonance (EPR) Analysis of Melanin Powders

The EPR spectra were obtained at 300 K using a commercial Bruker Biospin EMX (X-band) spectrometer (Billerica, MA, USA) operating at a frequency of 9.51 GHz. Several milligrams (20–25 mg) of the various melanin powder samples was put in 4 mm OD low-loss quartz tubes and inserted in the middle of the cylindrical microwave cavity. Typical microwave powers of 0.5–1 mW with 1 Gauss modulation amplitude and 100 kHz field modulation were employed for these experiments. The Zeeman splitting g-values of the signals were calibrated with the use of a DPPH (2,2-diphenyl-1-picrylhydrazyl) standard.

### 4.8. Thermogravitometric Analysis (TGA) of Char Yield

Char yield experiments were conducted using TGA on a TA Instruments TGA 5500 (New Castle, DE, USA), as previously described [34]. Samples were heated to 1000 °C at a constant rate (10 °C min^−1^) under a nitrogen purge (50 cm^3^ min^−1^). Thermograms were analyzed using TRIOS Thermal Analysis software (version 5.1) from TA instruments.

### 4.9. HEK293 Mammalian Cell Toxicity Test

The cytotoxicity of melanin to mammalian cells was determined using the CellTiter 96^®^ Aqueous One Solution MTS Cell Proliferation Assay (Promega, Madison, WI, USA). Briefly, HEK 293T/17 cells were seeded into 96-well tissue culture plates (~5 × 10^3^ cells/well in 100 µL media) and cultured for 24 h under standard incubation conditions. Next, 50 µL of DPBS (control) and DPBS containing increasing concentrations of melanin (stock solution 100 mg/mL; 10× serial dilutions) were added to the wells (in triplicate) and incubated for 2 h at 37 °C. After incubation, the melanin solutions were removed and the cells were washed with DPBS to remove the residual melanin. In total, 100 µL of complete growth medium was put into each well, and the cells were cultured for 48 h. After this proliferation period, the wells were read using a Tecan Spark Cyto plate reader for background absorption; then, 20 µL of tetrazolium substrate was added to each well and incubated at 37 °C for 3 h for formazan formation. The absorbance of the formazan product was read at 490 nm. The absorbance values of the formazan product were corrected by subtracting the background absorbance. The cell viability was calculated as a percentage of control cell proliferation (incubated with DPBS only) and plotted as a function of melanin concentration. The data were statistically analyzed using a two-way univariate analysis of variance (ANOVA). For multiple comparisons, Tukey’s test was applied.

## 5. Patents

A redox-inert, low-toxicity, high-stability melanin produced from DOPA by vanadate and selenite. Navy case number 212007 (6 February 2024).

## Figures and Tables

**Table 1:** Elemental composition (%).

	DOPA Melanin Control	Vanadate–DOPA Melanin	Selenite–Vanadate DOPA Melanin
carbon	64.99 ± 0.08	65.82 ± 1.20	64.62 ± 0.35
nitrogen	8.58 ± 0.22	7.57 ± 0.13	7.82 ± 0.08
oxygen	24.86 ± 0.13	25.24 ± 1.36	19.97 ± 0.07
sodium	0.16 ± 0.04	0.15 ± 0.04	2.50 ± 0.95
chlorine	1.43 ± 0.39	1.23 ± 0.33	4.50 ± 0.70
selenium	ND	ND	0.61 ± 0.08

ND indicates “Not detected”.

**Table 2:** Elemental ratios.

	DOPA Melanin Control	Vanadate–DOPA Melanin	Selenite–Vanadate DOPA Melanin
C/N	7.58 ± 0.18	8.70 ± 0.31	8.26 ± 0.13
C/O	2.61 ± 0.01	2.61 ± 0.19	3.24 ± 0.01
O/N	2.90 ± 0.06	3.34 ± 0.12	2.55 ± 0.04

**Table 3:** Elemental speciation within each group (%).

	DOPA Melanin Control	Vanadate–DOPA Melanin	Selenite–Vanadate DOPA Melanin
C-hydrocarbon	62.30 ± 0.85	59.15 ± 1.20	63.65 ± 1.20
C-heteroaromatic	24.85 ± 1.20	29.20 ± 1.13	23.70 ± 0.99
C-carboxylate	12.85 ± 0.35	11.65 ± 2.33	12.65 ± 0.21
O-hydroxyl	18.90 ± 1.27	17.10 ± 2.40	48.75 ± 4.03
O-carboxyl	81.10 ± 1.27	82.90 ± 2.40	51.25 ± 4.03
N-organic	92.40 ± 0.57	93.65 ± 0.78	96.00 ± 0.85
N-ammonium	7.60 ± 0.57	6.35 ± 0.78	4.00 ± 0.85
Cl-salt	45.05 ± 13.79	79.75 ± 2.47	55.00 ± 52.32
Cl-organic	54.95 ± 13.79	20.25 ± 2.47	45.00 ± 52.32

**Table 4:** Temperatures from TGA curves for degradation percent and maximal peak degradation.

Selenite (mM)	T (°C) 5%	T (°C) 25%	T (°C) 50%	T (°C) Peak
0	103.79 ± 18.06	328.47 ± 49.83	852.93 ± 3.18	915.75 ± 53.63
12.5	91.73 ± 4.92	314.42 ± 6.27	847.39 ± 14.39	211.56 ± 2.62
25	89.78 ± 7.46	320.09 ± 17.52	872.19 ± 7.82	84.14 ± 32.31
50	107.22 ± 5.18	302.63 ± 49.65	794.02 ± 29.04	856.69 ± 25.89

## Data Availability

The original contributions presented in this study are included in the article. Further inquiries can be directed to the corresponding author.

## Supplementary Materials

The following supporting information can be downloaded at https://www.mdpi.com/article/10.3390/ijms26125537/s1.

## Abbreviations

The following abbreviations are used in this manuscript:

ABTS	2,2′-azino-bis (3-ethylbenzothiazoline-6-sulfonic acid)
ANOVA	Analysis of variance
DHI	5,6-dihydroxyindole
DHICA	5,6-dihydroxyindole-2-carboxylic acid
DLS	Dynamic light scattering
DOPA	3,4-dihydroxyphenylalanine
DPBS	Dulbecco’s phosphate-buffered saline
DPPH	2,2-diphenyl-1-picrylhydrazyl
EPR	Electron paramagnetic resonance
FC	Ferrocene dimethanol
FRAP	Ferric reducing absorption power
LB	Lysogeny broth (also known as Luria–Bertani broth)
MM9v3	Marine M9 medium version 3
MTS	3-(4,5-dimethylthiazol-2-yl)-5-(3-carboxymethoxyphenyl)-2-(4-sulfophenyl)-2H-tetrazolium salt
PCA	Phenazine-1-carboxylic acid
SA:V	Surface area-to-volume
TEM	Transmission electron microscopy
UV-Vis	Ultraviolet-to-visible light spectrum
XPS	X-ray photoelectron spectroscopy
