# Supplementary material for: The Orthovanadate-Catalyzed Formation of a Thermally Inert and Low-Redox-Potential Melanin
Source: Int J Mol Sci. 2025 Jun 10;26(12):5537. doi: 10.3390/ijms26125537 (PMC12194018; doi:10.3390/ijms26125537)
Supplement: Supplementary file 1 [file ijms-26-05537-s001.zip › ijms-3635845-supplementary.pdf]

Supplemental Figures

Orthovanadate-catalyzed Formation of a Thermally Inert and Low Redox Potential Melanin

Eric VanArsdale [1], Olufolasade Atoyebi [2], Okhil Nag [1], Matthew Laskoski [2], Evan Glaser [3], Eunkeu Oh[4], Gary J. Vora [1] and Zheng Wang [1,*]

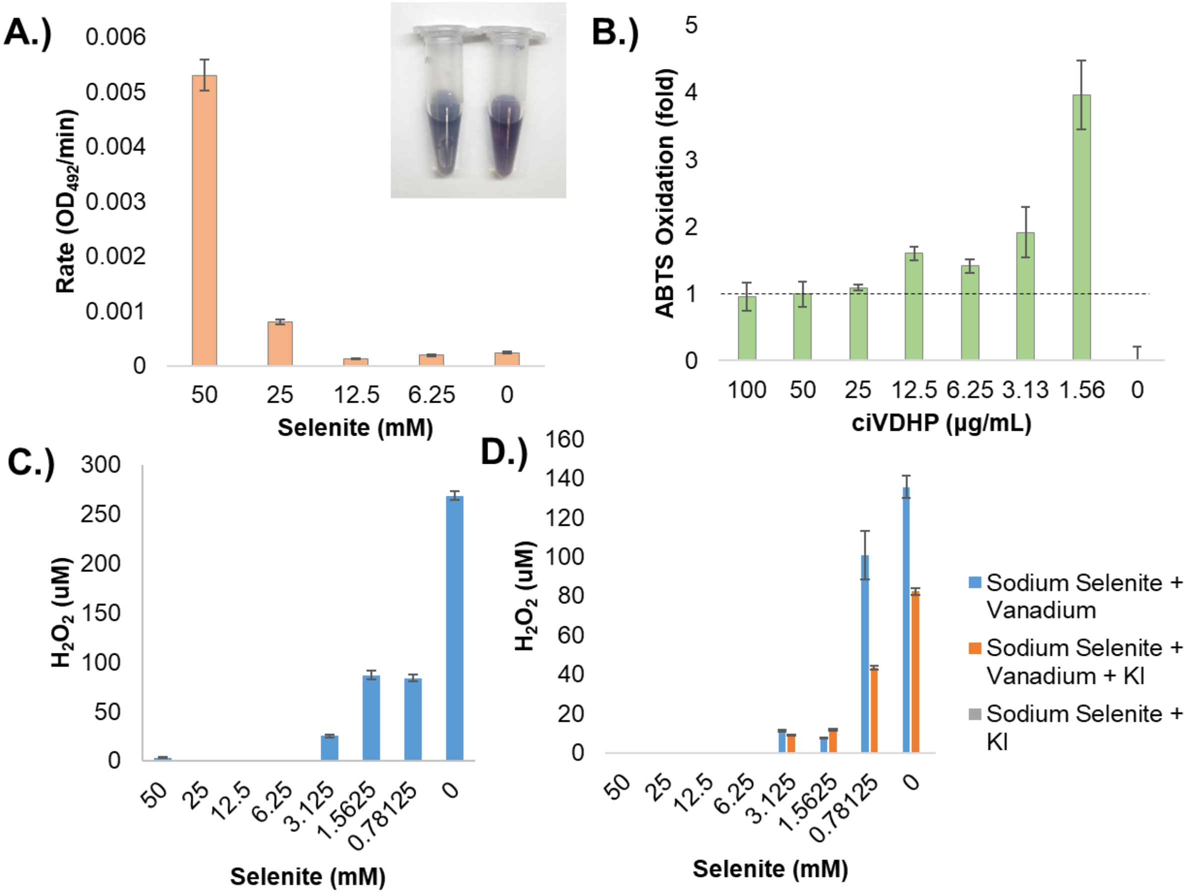

Figure S1. Chloroperoxidase discovery of non-enzymatic reaction. **A)** Calculated rate improvement from addition of purified ciVDHP enzyme (100 µg/mL) upon addition of selenite to buffer. Inset image shows the color change of 1 mM vanadate and 3 mM L-DOPA before (right) and after (left) addition of 50 mM selenite. **B)** Fold enhancement of ciVDHPO ABTS oxidation upon addition of 12.5 mM selenite. **C)** H_2O_2 measurements after overnight incubation of acetate buffer with serially diluted selenite. Selenite decreased H_2O_2 below measurable limits above 3.13 mM. **D)** H_2O_2 measurements after overnight incubation of acetate buffer with serially diluted selenite in the presence of 100 µM vanadate, 1 mM potassium or both.

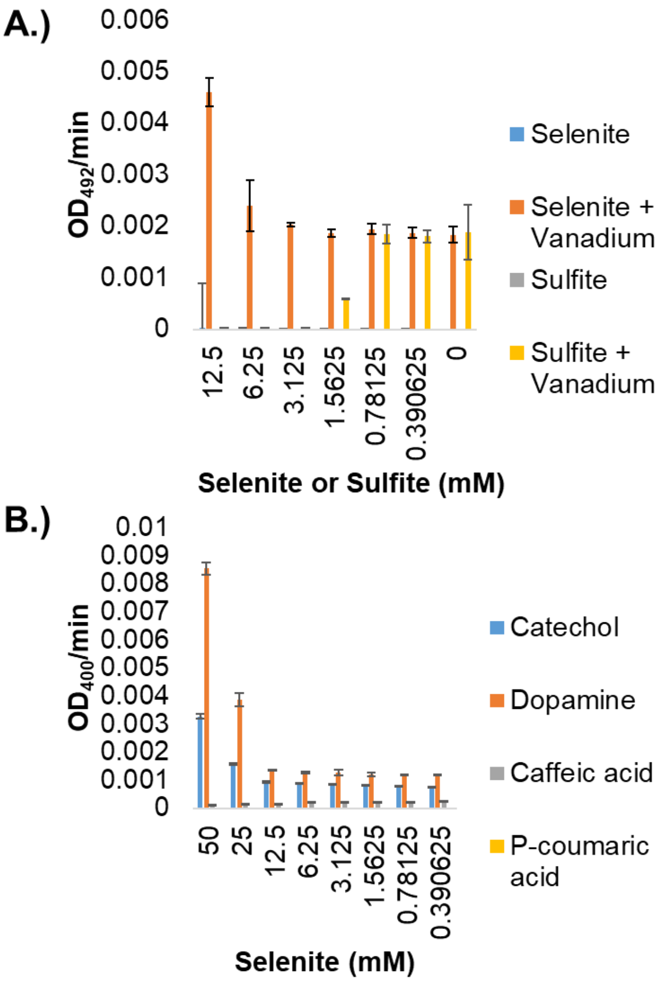

Figure S2. Dopachrome and melanin formation rate comparisons. **A)** The rate of melanin formation (OD_{492} measurements) in the presence of serially diluted selenite or sulfite with/ without 100 μM vanadium. **B)** The rate of melanin formation of 10 mM catechol, dopamine, caffeic acid, and p-coumaric acid in the presence of serially diluted selenite and 100 μM vanadium. P-coumaric acid did not form melanin. Caffeic acid was solubilized with 10% DMSO (v/v).

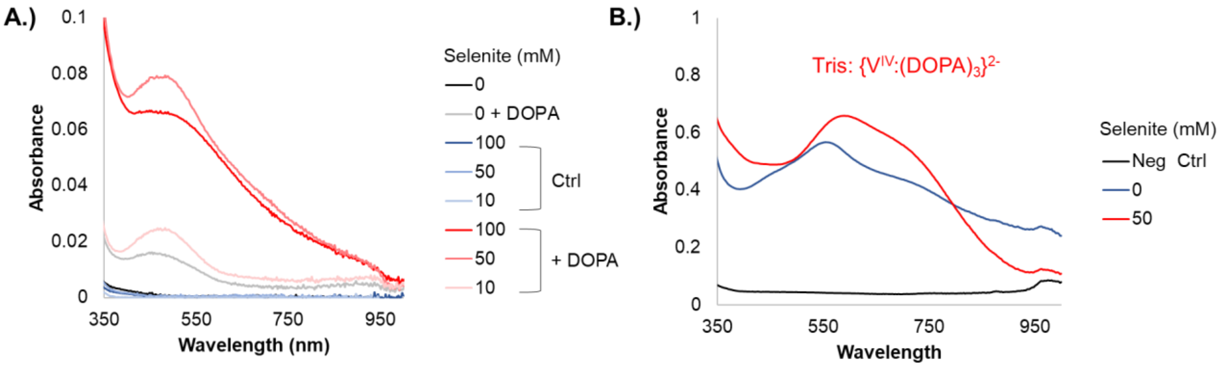

Figure S3. Spectral changes post-incubation. UV-Vis spectrum scans of various reaction conditions upon the addition of 15 mM DOPA to **A)** 100 μM vanadate or **B)** 1 mM vanadate with varying concentrations of selenite after 15 min of incubation.

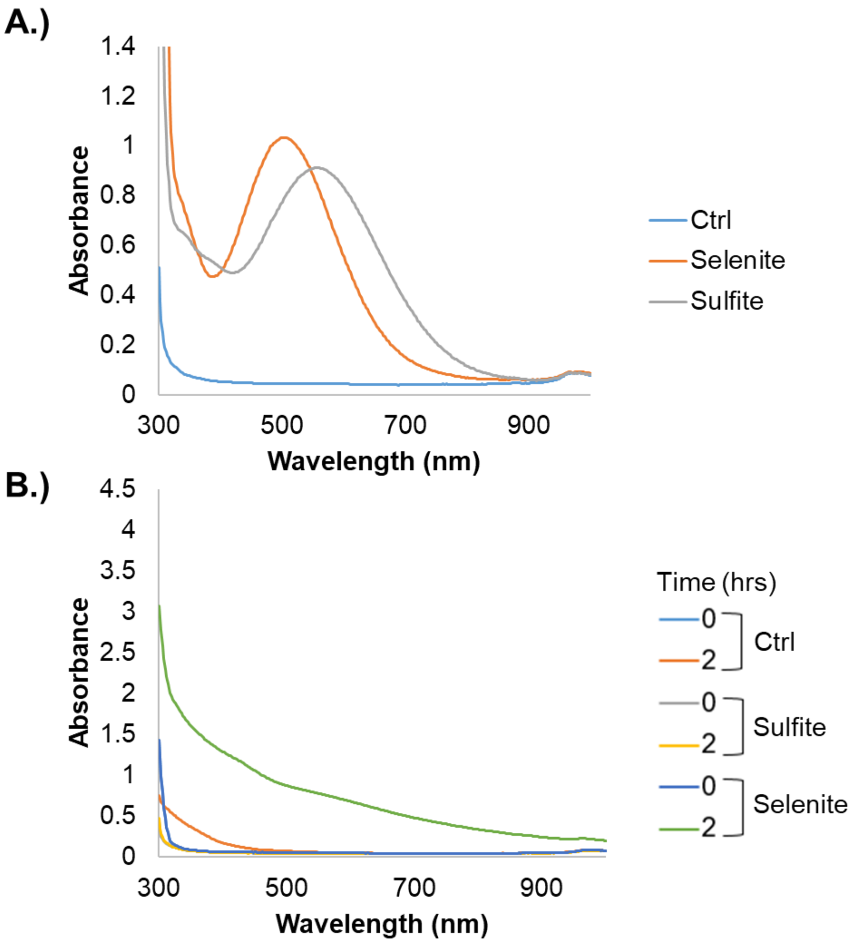

Figure S4. UV-Vis scans of ferric chloride with DOPA. **A)** UV-Vis spectrum of 1 mM ferric chloride and 15 mM DOPA in the presence of 100 mM selenite or sulfite. **B)** UV-Vis spectrum of 100 µM ferric chloride with 15 mM DOPA at 0 hr and 2 hr of incubation at 37 °C with 100 mM selenite or sulfite. All controls ("Ctrl") are 15 mM DOPA in acetate buffer (50 mM, pH 5.5).

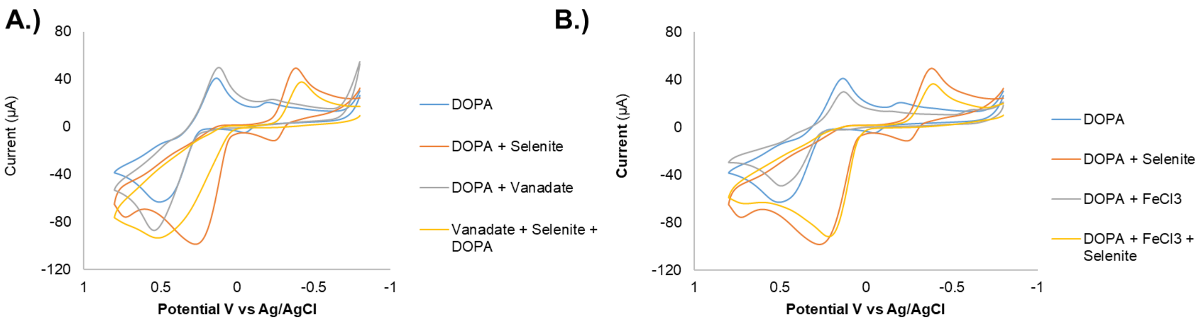

Figure S5. Cyclic voltammetry scans of metal-DOPA interactions with selenite. Cyclic voltammetry measurements of **A)** 1 mM vanadate and **B)** 1 mM ferric chloride with/without 100 mM selenite. All experiments exclude the first sweep which started at 0 V vs Ag/AgCl.

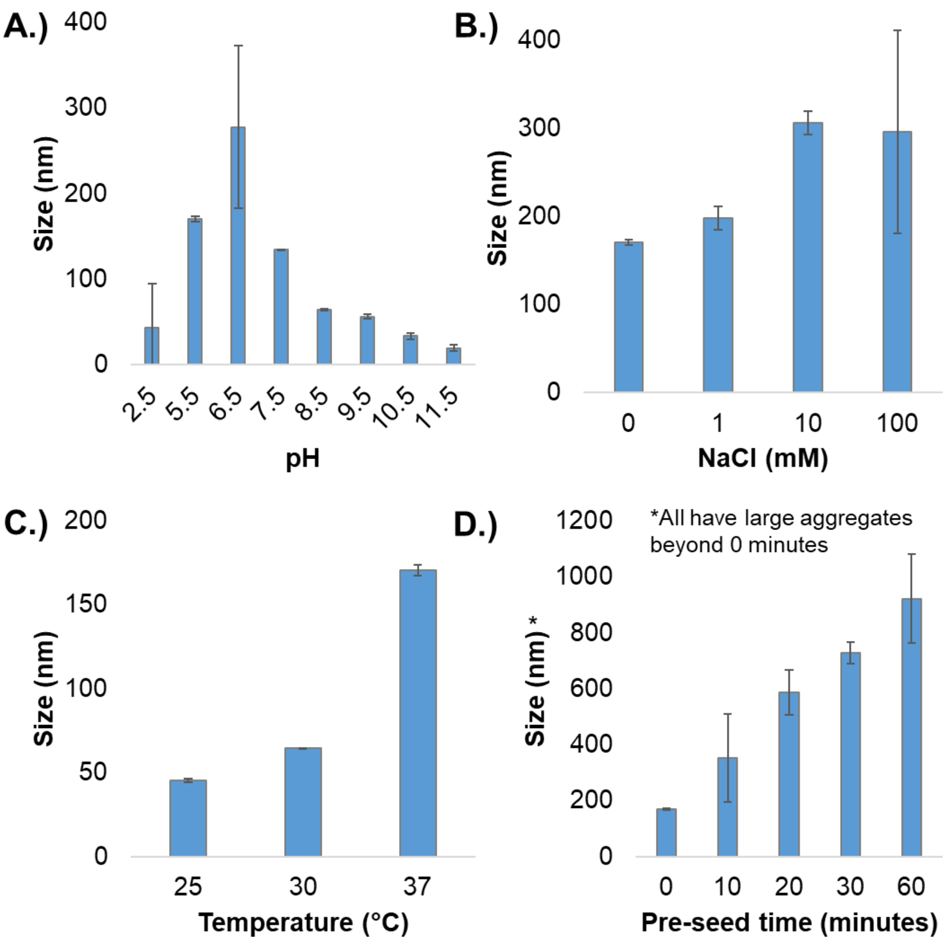

Figure S6. DLS measurements of selenite-melanin nanoparticles under biologically relevant conditions. **A-D)** DLS measurements of melanin nanoparticles formed after an overnight incubation. Unless indicated otherwise, the standard condition was pH 5.5, 0 mM NaCl, 37 °C, no pre-seed. Pre-seeding was performed by adding selenite after the designated time. All conditions were 50 mM selenite, 100 uM vanadate, 15 mM L-DOPA. Asterisks indicate the presence of large aggregates (>300 nm) with a broad distribution.

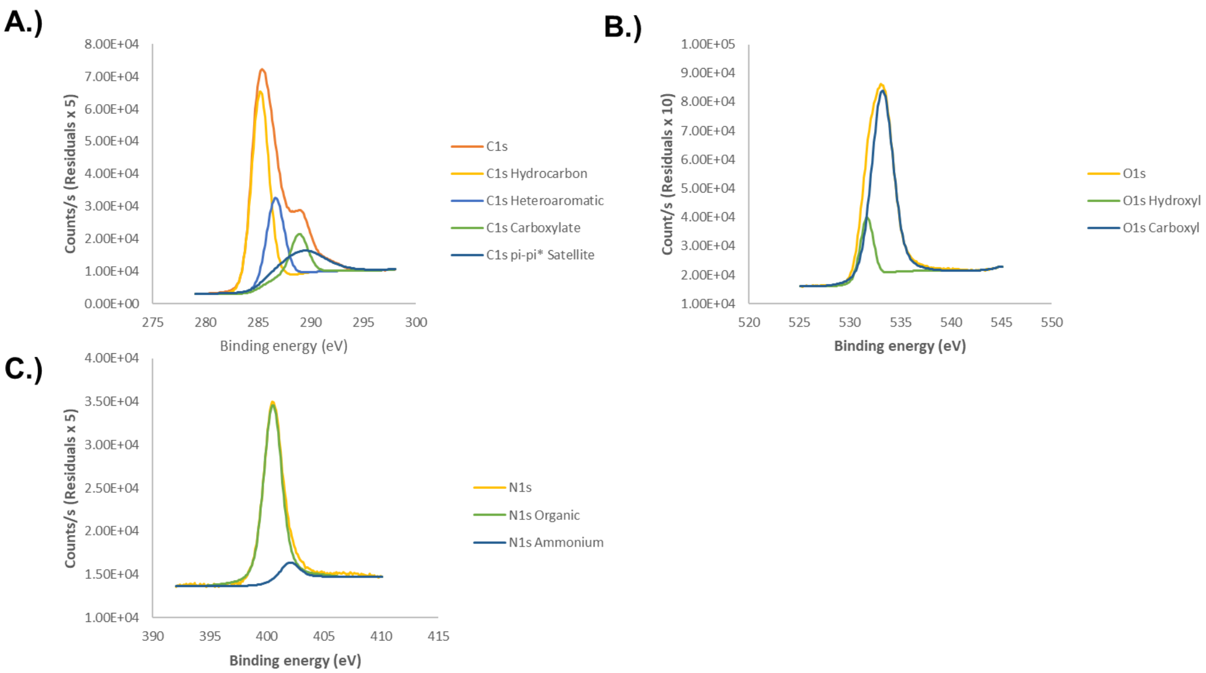

Figure S7. XPS analysis of DOPA-melanin. XPS scans of **A)** C1, **B)** O1, and **C)** N1.

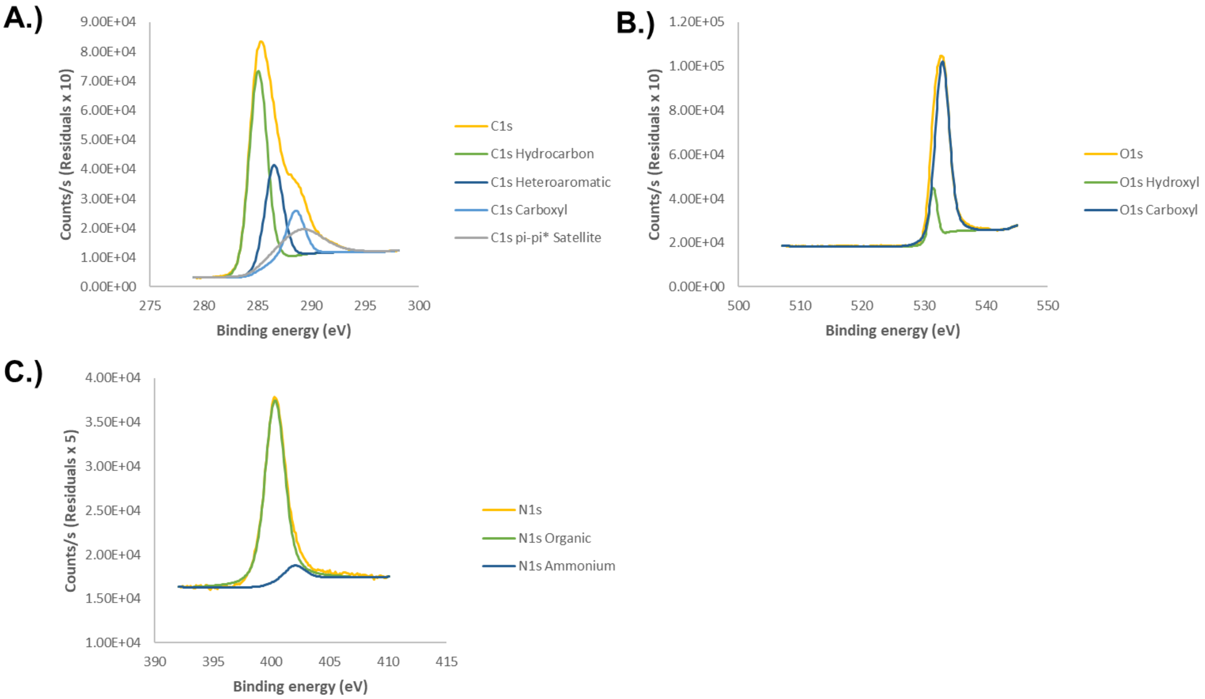

Figure S8. XPS analysis of vanadate-catalyzed DOPA-melanin. XPS scans of **A)** C1, **B)** O1, and **C)** N1.

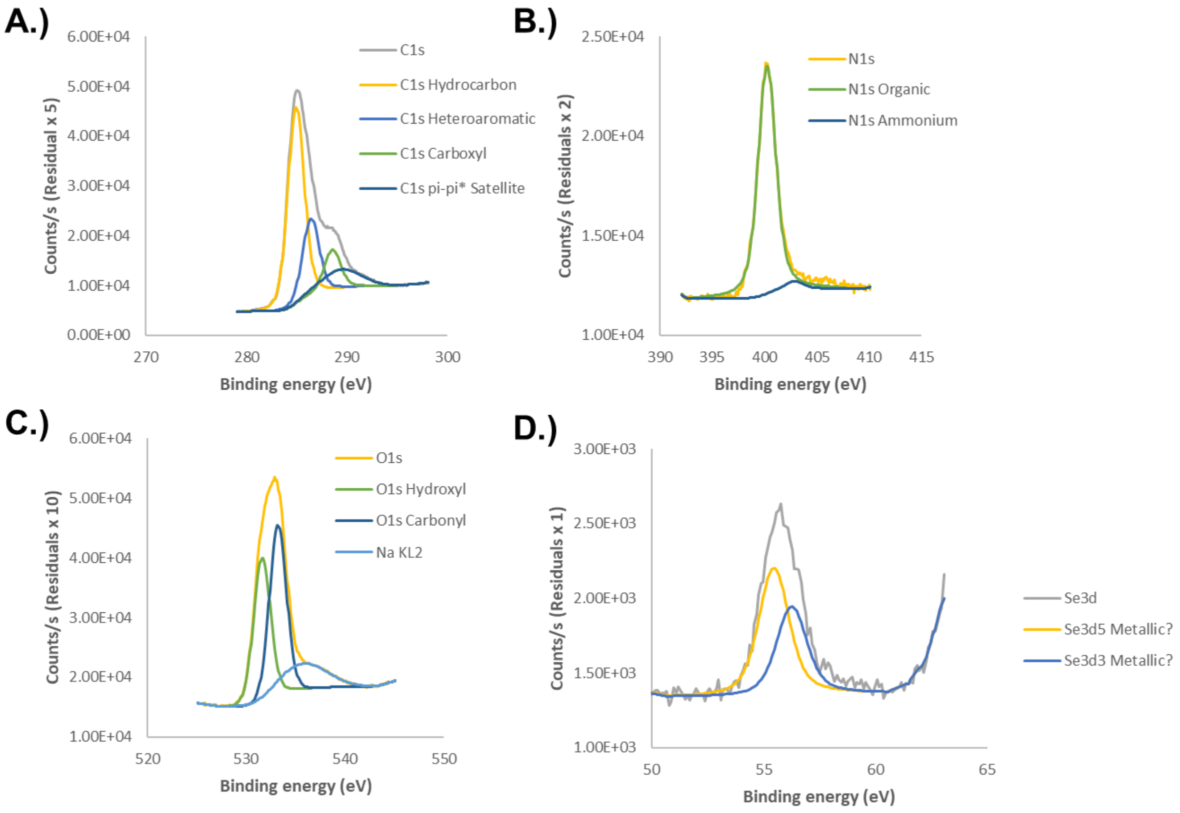

Figure S9. XPS analysis of vanadate-catalyzed DOPA-melanin with selenite. XPS scans of **A)** C1, **B)** O1, **C)** N1 and **D)** Se3d.

Table S1. XPS Peak Binding Energies (eV)

	Control melanin	Vanadate-DOPA melanin	Selenite-vanadate DOPA melanin
**Se3d5 Metallic?**	N/A	N/A	59.28 ± 5.37
**Se3d3 Metallic?**	N/A	N/A	63.08 ± 0
**Cl2p3 Salt**	197.98 ± 0	198.38 ± 0.14	198.38 ± 0.71
**Cl2p1 Salt**	199.68 ± 0	200.03 ± 0.07	200.03 ± 0.64
**Cl2p3 Organic**	199.33 ± 0.07	200.18 ± 0.14	198.83 ± 1.63
**Cl2p1 Organic**	201.03 ± 0.07	201.73 ± 0.07	205.88 ± 5.94
**C1s Hydrocarbon**	285.18 ± 0.14	284.93 ± 0.21	284.88 ± 0.14
**C1s Heteroaromatic**	286.63 ± 0.07	286.33 ± 0.35	286.38 ± 0.14
**C1s Carboxylate**	288.88 ± 0.14	288.33 ± 0.35	288.53 ± 0.07
**C1s pi-pi* Satellite**	289.48 ± 0	289.13 ± 0.21	289.83 ± 0.21
**N1s Organic**	400.43 ± 0.07	400.18 ± 0.14	400.18 ± 0.14
**N1s Ammonium**	401.88 ± 0.28	402.03 ± 0.07	402.48 ± 0.57
**O1s Hydroxyl**	531.58 ± 0.14	531.33 ± 0.21	531.63 ± 0.07
**O1s Carboxyl**	533.13 ± 0.07	532.88 ± 0.14	533.13 ± 0.07
**Na KL2**	N/A	N/A	535.33 ± 0.92
**Na1s Salt**	1072.18 ± 0	1071.83 ± 0.07	1072.58 ± 0.42

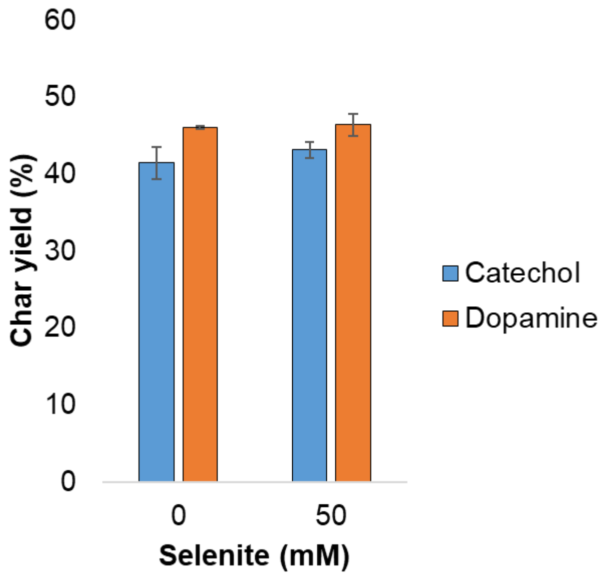

Figure S10. TGA assessment of selenite-melanin formed from catechol and dopamine. Char yield of TGA analysis of 10 mM catechol and dopamine melanin powders with/without selenite.
